# Advanced Image Segmentation and Modeling – A Review of the 2021–2022 Thematic Series

**DOI:** 10.1186/s41205-022-00163-7

**Published:** 2023-01-24

**Authors:** Prashanth Ravi

**Affiliations:** Department of Radiology, University of Cincinnati College of Medicine, Cincinnati, OH USA

**Keywords:** Medical 3D printing, Additive manufacturing, Image segmentation, Medical devices, Point of care, Augmented reality, Quality assurance

## Abstract

Medical 3D printing is a form of manufacturing that benefits patient care, particularly when the 3D printed part is patient-specific and either enables or facilitates an intervention for a specific condition. Most of the patient-specific medical 3D printing begins with volume based medical images of the patient. Several digital manipulations are typically performed to prescribe a final anatomic representation that is then 3D printed. Among these are image segmentation where a volume of interest such as an organ or a set of tissues is digitally extracted from the volumetric imaging data. Image segmentation requires medical expertise, training, software, and effort. The theme of image segmentation has a broad intersection with medical 3D printing. The purpose of this editorial is to highlight different points of that intersection in a recent thematic series within *3D Printing in Medicine*.

## Introduction

Medical 3D printing uses specialized segmentation and computer-aided design software [1, 2], and there are society guidelines [3] that recommend that these software be cleared by the United States Food and Drug Administration when the clinical service is performed in the United States [3]. At the University of Cincinnati Radiology 3D Printing Lab, segmentation and computer-aided design are performed using the Materialise Mimics Innovation Suite, software cleared by the United States Food and Drug Administration, as part of the routine service for all patient specific anatomic models and guides. Image segmentation plays a critical role in medicine [4, 5] and is a natural pre-cursor to digital modeling and the 3D printing of anatomic models and related medical devices [1, 6]. The “Advanced Image Segmentation and Modeling” thematic series highlights cutting-edge applications of image segmentation for medical training, interventional planning, low-cost medical device development, augmented reality, and quality assurance [7]. These applications reflect the evolving landscape of medical 3D printing. As peer-reviewed evidence continuous to build surrounding the technology led by the American College of Radiology’s 3D Printing Registry [8], it is likely that reimbursement using Current Procedure Terminology I codes could become a reality in the near to medium term [2, 8, 9]. This could be a pivotal moment for medical 3D printing that results in subsequently exponential adoption of the technology across the United States due to the unlocked revenue stream. The increased engagement would likely result in removal of many of the technological bottlenecks currently plaguing the otherwise powerful technology. The future of medical 3D printing looks promising and this collection of articles is a reflection of the exciting advancements and other applications that lie ahead.

## Training and Simulation

**Fig. 1:**
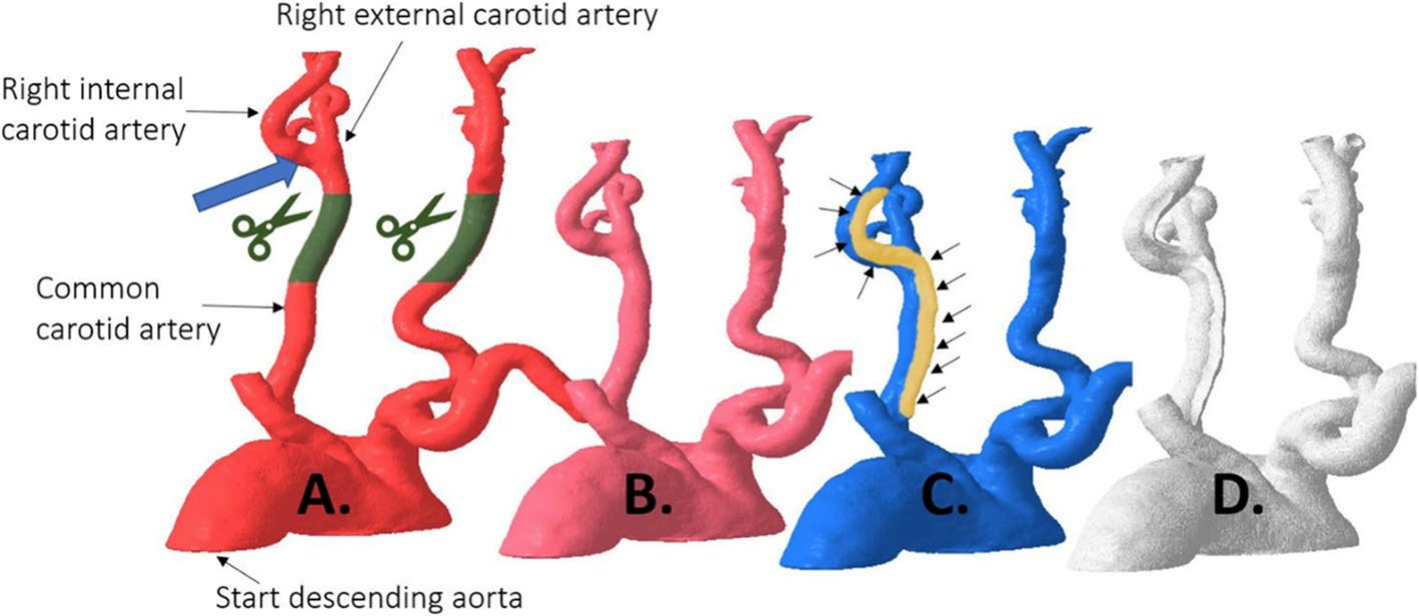
The process of 3D printing a deroofed carotid artery model for stent-deployment training as described by De Backer et al. [10]

Visual feedback during stent-deployment is impossible to obtain as deployment is performed under fluoroscopic imaging. Using 3D printed models, De Backer et al. fabricated patient-specific anatomies (Fig. 1) for stent-deployment training and for patient education [10]. The deroofed model allowed clear visualization of the bottlenecks and features of carotid artery stent deployment without the need for fluoroscopic guidance.

**Fig. 2:**
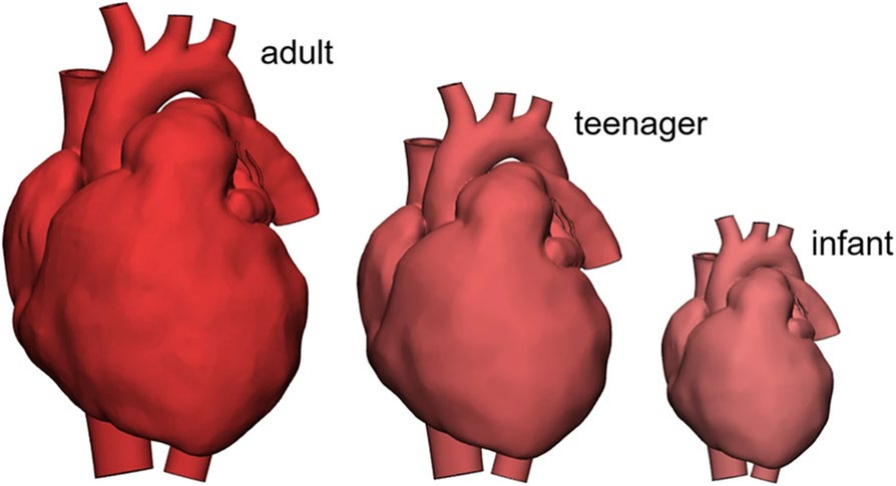
The scaling process developed by Hopfner et al. for young heart models using adult patient scans [11]

In small children, both CT and MRI imaging is rare since minimization of radiation and sedation is important. Hopfner et al. used image processing and computer-aided design software to allow unlimited variations of 3D heart models based on single patient scans [11]. The adult heart was scaled to 80% for simulating a teenage heart and to 55% for simulating an infant heart (Fig. 2). The authors created 4 example models using trimming, cutting, hole editing, and other tools. All models were successfully used in teaching or hands-on training courses.

**Fig. 3:**
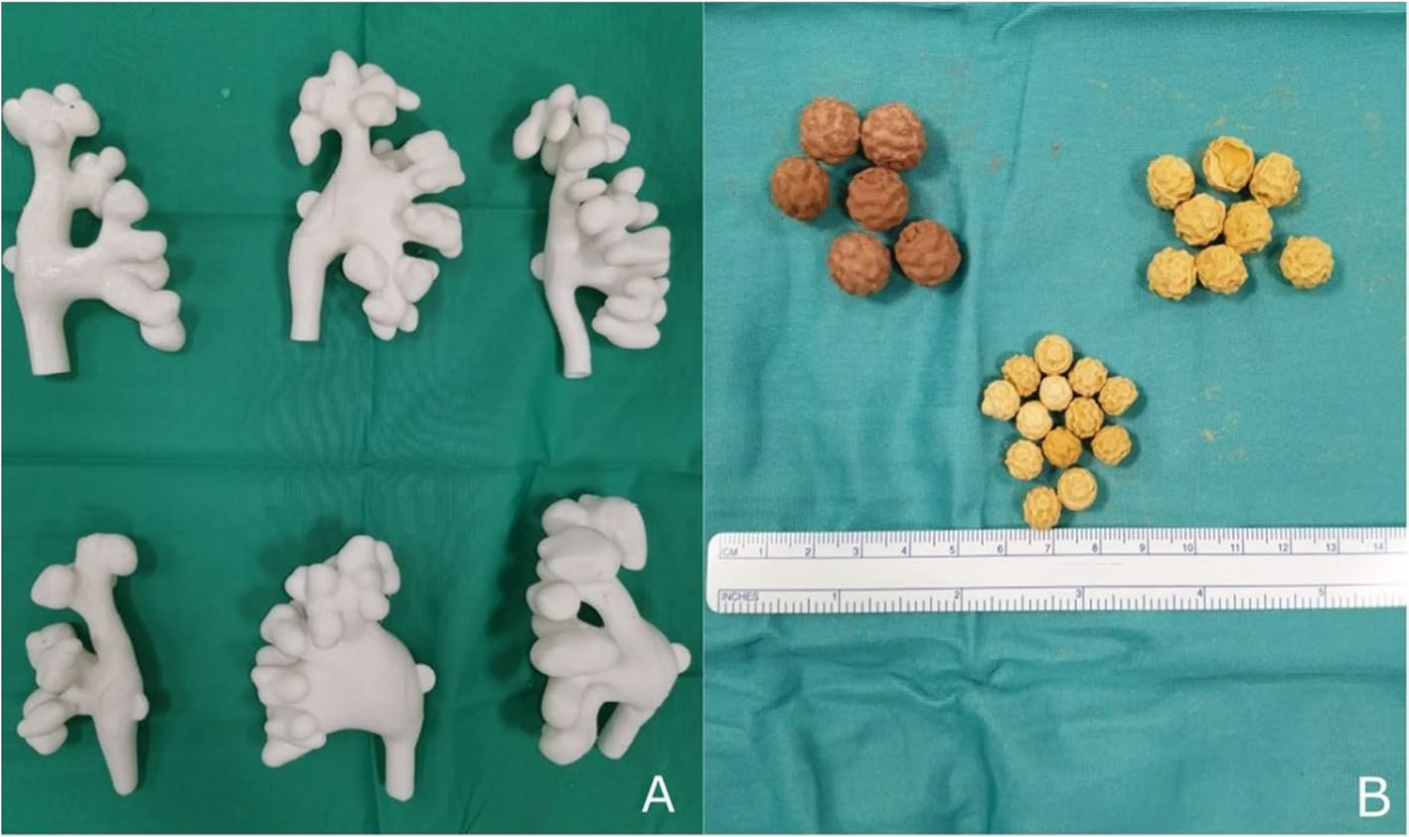
(A) Three-dimensional printed training models of different pelvicalyceal systems and (B) training stones developed by Orecchia et al. [12]

Medical training in retrograde intrarenal surgery for treating renal stone disease is arduous owing to the complexity of the procedure. A series of six 3D printed models of upper urinary tract and stones (Fig. 3) were developed by Orecchia and colleagues for improving the training process [12]. The molds for the stones were developed using 3D printing and soft as well as hard stones in different sizes were produced from these molds. The models match incredible anatomical resemblance with low production cost and high reusability.

**Fig. 4:**
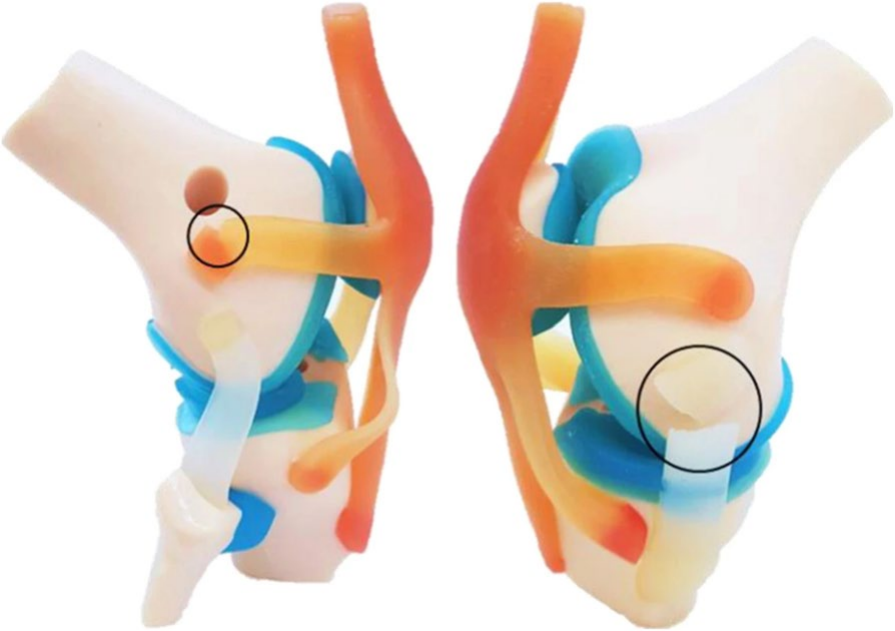
Full color knee joint model developed by Ruiz et al. [13]

Full color and realistic joint models can be valuable for studying complex cases. A new method for developing multi-color and multi-material life-like knee joint anatomical models (Fig. 4) was developed by Ruiz et al. [13]. Using different computer-aided design systems and material jetting 3D printing, three anatomical models were 3D printed with mimicry of fibrous matrix. The proposed models could be considered as alternatives to cadaveric specimens for medical training.

## Interventional Planning

Single field orthovoltage radiation has dosimetric pitalls and unnecessarily excessive exposure of radiation to organs at risk. Cheng et al. present a novel technique incorporating an optical scanner and 3D printing to deliver treatments using parallel opposed fields [14]. A retrospective review of 26 patients treated with this technique between 2015 and 2019 was undertaken. An optical scan of the face was first performed, and the positive impressions were 3D printed. Custom 4 cm thick nose block boluses were made with wax encased in a acrylic shells using the 3D printed face models. The complete response rate at a median follow-up of 6-months was 88% with 1 patient having a refractory tumor and 1 having a recurrence. Use of 3D printing with parallel opposed fields allowed an effective treatment of carcinomas of the nose with high control rate and low toxicity profiles.

## Medical Devices

**Fig. 5:**
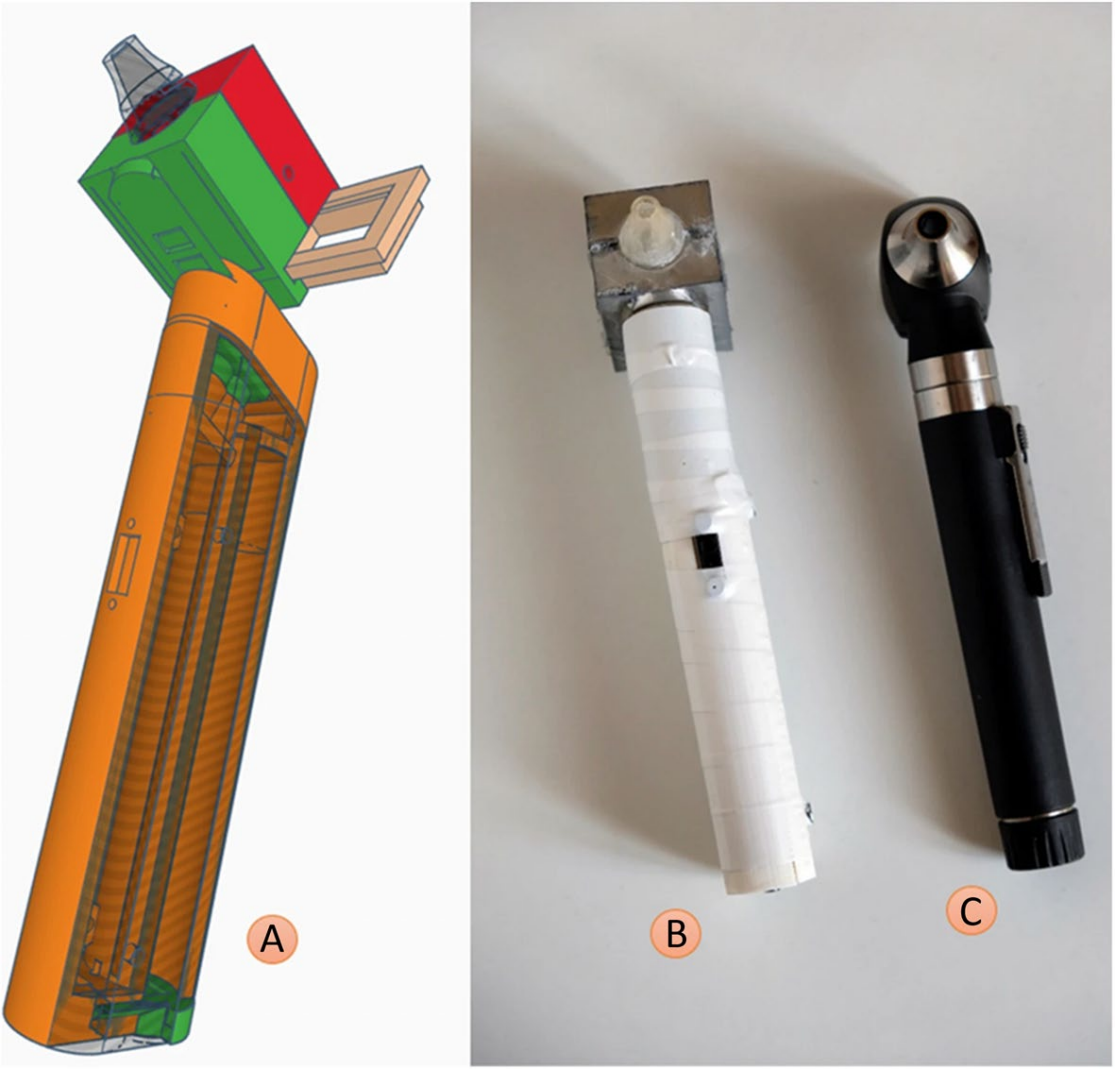
Computer-aided design and 3D printed model of an otoscope developed by Capobussi et al. along with a commercial otoscope [15]

Limited access to key diagnostic tools is detrimental to priority health needs of populations. In situations where an otoscope is unavailable due to financial constraints, a self-fabricated low-cost otoscope might represent a feasible opportunity. Capobussi et al. share the design and development of an open-source 3D printed otoscope and the prototype is compared to a commercial solution (Fig. 5) demonstrating similar overall quality between the instruments [15].

## Augmented reality

**Fig. 6:**
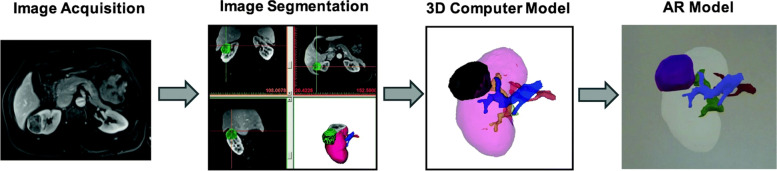
Augmented Reality workflow developed by Wake et al. [16]

Visualizing patient-specific three-dimensional imaging data in augmented reality may improve the surgeon’s understanding of anatomy and surgical pathology, thereby allowing for improved surgical planning, superior intra-operative guidance, and ultimately improved patient care. Wake et al. developed a workflow using the Microsoft Hololens device to visualize prostate and renal cancer models (Fig. 6) to guide surgery [16].

## Quality Assurance

Sterilization of a 3D printed model could negatively impact its geometric fidelity. The sterility, biocompatibility, mechanical properties, and geometric fidelity of anatomic models must be carefully considered. Toro et al. investigated the geometric fidelity of material extrusion 3D printed acrylonitrile butadiene styrene polymer using vaporized hydrogen peroxide sterilization [17]. Models from 16-patient CT scans were studied and the dimensional error of the sterilized parts compared to the original designs were − 0.082 mm for the models and 0.126 mm for the guides. The dimensional stability of both the models and guides was not affected after low-temperature sterilization with vaporized hydrogen peroxide. Three-dimensional printed saw guides are often used to improve osteotomy results and are generally designed using CT imaging despite the radiation burden. Willemsen et al. investigated the usability of MR-based synthetic-CT imaging for the design and 3D printing of patient-specific saw guides [18]. A similar error was found when comparing synthetic-CT and CT digital surface models to ground truth micro-CT models. Moreover, the saw guide placement errors were also equivalent.

## Summary

The collection of articles displayed a diverse set of cutting-edge applications that spanned medical training, interventional planning, medical devices, augmented reality, and quality assurance. This is only a subset of the potential pool of medical 3D printing applications, but still reflects the potential spectrum of the technology.

## Data Availability

Not applicable.
